# A Synergistic Role of Photosynthetic Bacteria and Fungal Community in Pollutant Removal in an Integrated Aquaculture Wastewater Bioremediation System

**DOI:** 10.3390/biology14080959

**Published:** 2025-07-30

**Authors:** Muhammad Naeem Ramzan, Ding Shen, Yingzhen Wei, Bilal Raza, Hongmei Yuan, Arslan Emmanuel, Zulqarnain Mushtaq, Zhongming Zheng

**Affiliations:** 1 School of Marine Sciences, Ningbo University, Ningbo 315211, China; naeem2726896@gmail.com (M.N.R.); sd604038720@163.com (D.S.); weiyingzhen1998@163.com (Y.W.); balirana321@gmail.com (B.R.); hongmei.yuan@hotmail.com (H.Y.); arslanemmanuel88@gmail.com (A.E.); 2 Ningbo China Institute for Supply Chain Innovation—MIT Global Scale Network, Ningbo 315066, China; zulqarnainmushtaq@yahoo.com; 3 School of Economics and Finance, Jiangxi University of Finance and Economics, Nanchang 330032, China

**Keywords:** aquaculture wastewater, FUNGuild, fungal community, bioremediation, ITS sequencing

## Abstract

This study addresses the understanding of fungal diversity and their bioremediation roles in an integrated aquaculture wastewater bioremediation system, an area less explored compared to bacteria, viruses, and protozoa. Despite the rapid advancement and affordability of molecular tools, insights into fungal communities remain vague, and interpreting environmental studies in an ecologically meaningful manner continues to pose challenges. To bridge this knowledge gap, we developed an integrated aquaculture wastewater bioremediation system, incorporating photosynthetic bacteria, and utilizing internal transcribed spacer (ITS) sequencing to analyze fungal community composition. Our findings indicate that the fungal community in aquaculture wastewater is predominantly composed of the phyla Ascomycota and Chytridiomycota, with dominant genera including *Aspergillus*, *Hortea*, and *Ciliphora*. FUNGuild, a user-friendly trait and character database operating at the genus level, facilitated the ecological interpretation of fungal functional groups. The analysis revealed significant negative correlations between nutrient levels (COD_mn_, NH_4_^+^-N, NO_3_^−^-N, NO_2_^−^-N, and PO_4_^−3^-P) and specific fungal functional groups, including epiphytes, animal pathogens, dung saprotrophs, plant pathogens, and ectomycorrhizal fungi. The removal rate for the COD_mn_, NH_4_^+^-N, NO_3_^−^-N, NO_2_^−^-N, and PO_4_^−3^-P were 71.42, 91.37, 88.80, 87.20, and 91.72% respectively. This study highlights the potential role of fungal communities in bioremediation processes and provides a framework for further ecological interpretation in aquaculture wastewater treatment systems.

## Simple Summary

This study explores the largely overlooked role of fungal communities in aquaculture wastewater bioremediation systems. Using an integrated treatment setup enhanced with photosynthetic bacteria and internal transcribed spacer (ITS) sequencing, the research reveals that Ascomycota and Chytridiomycota dominate the fungal community, with key genera such as *Aspergillus*, *Hortea*, and *Ciliphora*. Functional group analysis via FUNGuild showed significant negative correlations between nutrient levels and several fungal groups, indicating their involvement in pollutant reduction. The system achieved removal rates of 71.42% (CODMn), 91.37% (NH_4_^+^-N), 88.80% (NO_3_^−^-N), 87.20% (NO_2_^−^-N), and 91.72% (PO_4_^3−^-P). These findings underscore the ecological significance of fungi in wastewater treatment and offer a foundation for future research on their functional roles in such systems.

## 1. Introduction

The aquaculture industry represents one of the most rapidly expanding sectors in global food production, accounting for approximately 47% of the fish consumed by humans. With global population growth, aquaculture output is projected to increase by 60% to 100% over the next two to three decades to meet rising demand [1,2]. However, the intensification of aquaculture practices leads to increased input requirements and, consequently, elevated waste generation. Effluents from aquaculture systems are typically rich in suspended solids, organic matter, nitrogen, and phosphorus, which, when discharged into natural water bodies, contribute to nutrient pollution, eutrophication, and degradation of aquatic ecosystems [1]. To mitigate the environmental impacts of aquaculture effluents, biological wastewater treatment systems have become indispensable. These systems are designed to maintain water quality by minimizing the release of nutrients and pollutants into surrounding ecosystems [3]. Among biological treatment methods, bioremediation has gained prominence as a sustainable approach, utilizing living organisms such as plants, bacteria, fungi, and algae to degrade or transform toxic substances into less harmful forms. Microorganisms, due to their metabolic flexibility and adaptability, play a pivotal role in this process [4]. Photosynthetic bacteria (PSB) have emerged as a promising group of microorganisms for bioremediation applications. These bacteria have been effectively utilized in environmental waste control, ecological restoration, agriculture, aquaculture, and even in the chemical and pharmaceutical industries, offering both environmental and economic benefits [5,6]. Since the early studies on PSB in wastewater treatment were initiated in 1971 [7], numerous investigations have confirmed their efficacy in nutrient removal and pollutant degradation. PSB exhibits unique capabilities, including phototrophic growth, CO_2_ fixation, and the oxidation of compounds such as taurine under specific conditions [8]. Moreover, the metabolic processes of PSB not only enhance wastewater treatment efficiency but also generate valuable by-products, such as carotenoids, which have applications in the food and cosmetics industries [9,10,11]. Photosynthetic bacteria (PSB), including *Rhodopseudomonas*, *Rhodobacter*, and *Rhodococcus*, have been extensively utilized for pollutant treatment. *Rhodopseudomonas* plays a pivotal role in wastewater management by efficiently mineralizing organic waste, removing hydrogen sulfide, and participating in denitrification processes [12]. It has been demonstrated to promote the growth of purple non-sulfur bacterial biomass in various wastewater sources, thereby reducing chemical oxygen demand (COD) and lowering concentrations of various ions and compounds [13]. Furthermore, strains of *Rhodopseudomonas* have been shown to enhance aquaculture water quality, boost disease resistance, and improve the growth and yield of fish species [14]. *Rhodococcus*, on the other hand, plays a critical role in pollutant treatment due to its exceptional biodegradation capabilities and diverse metabolic pathways [15,16]. However, the treatment efficacy of single bacterial strains can be limited in practical applications. Composite strains offer significant advantages, particularly in addressing complex contaminants [17]. For instance, a reagent composed of *Rhodobacter blasticus* and *Rhodobacter capsulatus* in a 1:1 ratio was shown to reduce COD in anaerobically digested swine wastewater by 83.3%, which was 19.3% higher than using *Rhodobacter blasticus* and 10.6% higher than using *Rhodobacter capsulatus* individually. Additionally, a compound of *Bacillus cereus*, *Bacillus amyloliquefaciens*, and *Pseudomonas stutzeri* proved effective in reducing ammonium nitrogen (NH_4_^+^-N), nitrate (NO_3_^−^-N), and nitrite (NO_2_^−^-N) in aquaculture wastewater [18]. These findings collectively underscore the significant potential of PSB in bioremediation strategies to address environmental pollution challenges.

In parallel with bacterial bioremediation, fungi have been recognized for their role in environmental detoxification through mycoremediation. This process employs specific fungi, particularly filamentous fungi and mushrooms, to degrade organic pollutants and remediate contaminated environments [19]. Despite their potential, the application of fungi in aquaculture wastewater treatment has received less attention compared to bacteria, primarily due to challenges such as competition with indigenous microbial communities and the need for continuous inoculation to sustain remediation processes [20]. Fungi are metabolically versatile organisms capable of producing a wide array of extracellular enzymes, which facilitate the degradation of complex organic compounds [21]. They play a crucial role in nutrient cycling and organic matter decomposition, contributing to the overall health and stability of aquatic ecosystems. Eukaryotic microorganisms, such as members of the Cafeteriaceae, Saccharomycetales, and Agaricomycetes, have been shown to dominate various wastewater treatment stages, underscoring their importance in bioremediation processes [22]. Furthermore, fungal biomass can yield bioactive compounds with antioxidant, antimicrobial, immunostimulant, and hypoglycemic properties, offering additional economic benefits [23,24]. Our previous studies have primarily focused on the role of PSB in modulating bacterial communities and enhancing nutrient removal in aquaculture wastewater systems [18]. However, the interactions between PSB and fungal communities, and their combined effects on wastewater treatment efficiency, remain underexplored. This study aims to address this knowledge gap by investigating the structural and functional changes in fungal communities in response to PSB addition in aquaculture wastewater. Additionally, we assessed how these changes contribute to nutrient removal, providing new insights into the synergistic potential of PSB and fungi in bioremediation strategies for sustainable aquaculture.

## 2. Materials and Methods

### 2.1. Experimental Setup

**Figure 1:**
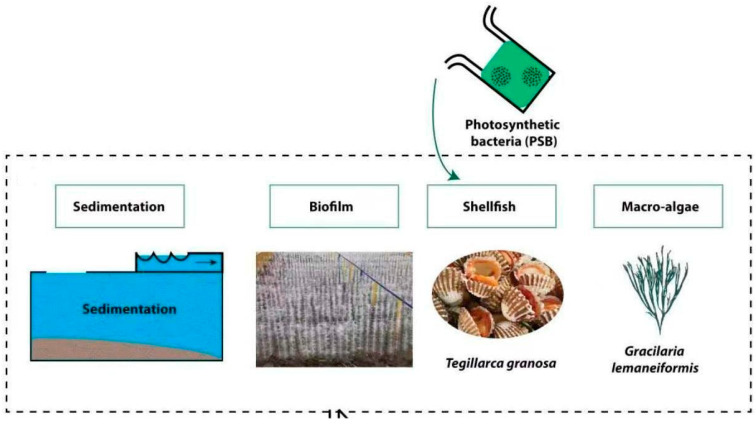
The graphical representation of the integrated aquaculture wastewater bioremediation system (IAWBS).

This research was carried out at the pilot test base of Ningbo University, located in Meishan, where an integrated aquaculture wastewater bioremediation system (IAWBS) was implemented (Figure 1). The IAWBS comprised four sequential treatment units: sedimentation, biofilm, filter-feeding shellfish, and macroalgae. Each unit was contained within a 70 L white polyethylene (PE) barrel, with wastewater flowing progressively through the system. The biofilm unit was equipped with polyethylene brushes (0.2 m in diameter and 0.5 m in length), installed at a density of four brushes per barrel. To ensure complete submersion, one end of each brush was fixed to the barrel’s base, while the other end was suspended vertically at the center. The shellfish unit was populated with *Tegillarca granosa* clams at a stocking density of 70 individuals per square meter, adhering to the protocol outlined by Nicholaus et al. (2019) [25]. In the macroalgae unit, *Gracilaria lemaneiformis* was introduced at a concentration of 0.2 g per liter. The wastewater utilized in the experiment was obtained from a high-density greenhouse shrimp farm (Haohai Aquaculture Farm) in Yinzhou District, Ningbo City, Zhejiang Province, China. The pH of the water was 7.8~8.2, and the temperature was maintained between 24 and 26 °C, and HRT was 14 days. To replicate the wastewater treatment process, 20 L of wastewater was circulated through the treatment units every two days in the following order: sedimentation → biofilm → shellfish → macroalgae. A microbial consortium consisting of *Rhodopseudomonas palustris*, *Rhodobacter capsulatus*, and *Rhodospirillum rubrum* (collectively referred to as 3Rs) was prepared for the study. The consortium was cultivated in a light incubator at 30 °C under a light intensity of 3000–4000 LX to activate the bacterial strains. In the treatment group, 1‰ photosynthetic bacteria (PSB), specifically the 3Rs consortium, were introduced into the shellfish unit every four days. The 3Rs strains were combined in a ratio of 2:2:3, as supplied by the BeNa Culture Collection Center. The experimental trial was conducted over a period of one month. 

### 2.2. Sample Collection and Measurement Method

The aquaculture wastewater used in this study primarily consisted of effluents discharged from mariculture systems, which are typically rich in organic matter and dissolved nutrients. Key components of the wastewater included ammonium (NH_4_^+^-N), nitrate (NO_3_^−^-N), nitrite (NO_2_^−^-N), orthophosphate (PO_4_^3−^-P), organic matter, and other constituents. Water and microbial samples were collected every two days over the course of the 30-day experimental period. At each sampling point, water was thoroughly mixed by collecting from the top, middle, and bottom of the water column to ensure a representative composite sample. A total of 500 mL of water was pooled from individual replicate samples to create a composite sample representing each treatment condition. From this pooled volume, 100 mL was used for microbial filtration. For water quality analysis, two types of samples were collected: Filtered samples (using 0.45 μm polycarbonate membranes) were used to analyze dissolved nutrients such as ammonia (NH_4_^+^-N), nitrite (NO_2_^−^-N), nitrate (NO_3_^−^-N), and phosphate (PO_4_^3−^-P). Unfiltered samples were used for the determination of total nitrogen (TN), total phosphorus (TP), and chemical oxygen demand (COD_mn_). Samples for microbial community analysis were filtered through 0.22 μm polycarbonate membranes using a vacuum filtration system. The filters were immediately stored at −80 °C until DNA extraction. All collected water samples were stored in pre-cleaned polyethylene bottles, kept on ice during transport, and then stored at −20 °C until analysis. Each treatment group consisted of three biological replicates, and consistent sample handling protocols were followed to ensure data reliability.

NH_4_^+^-N, NO_3_^−^-N, NO_2_^−^-N, PO_4_^3−^-P, TN, and TP were quantified using an automatic discontinuous chemical analyzer (SmartChem, London, UK). Specifically, NH_4_^+^-N, TN, TP, NO_3_^−^-N, NO_2_^−^-N, PO_4_^3−^-P, and COD_Mn_ were measured using hypobromite oxidation, potassium persulfate oxidation, cadmium column reduction, naphthalene ethylenediamine spectrophotometry, phosphomolybdenum blue spectrophotometry, and alkaline potassium permanganate methods, respectively [26].

The concentration of NH_4_^+^-N was quantified using hypobromite oxidation [27]. For NO_3_^−^-N, the content was determined by reduction via a cadmium column [28]. The NO_2_^−^-N concentration was measured spectrophotometrically using the naphthylethylenediamine method [18]. Additionally, PO_4_^3−^-P content was analyzed spectrophotometrically through the phosphomolybdenum blue method [29].

### 2.3. Internal Transcribed Spacer Sequencing and Bioinformatics Analysis

DNA extraction was performed using the minkgene Water DNA Kit, and the concentration and purity of the extracted DNA were assessed using a NanoDrop One spectrophotometer (Thermo Fisher Scientific, Waltham, MA, USA). The ITS1 and ITS2 region of fungal DNA was amplified using specific primers: forward primer (GCATCGATGAAGAACGCAGC) and reverse primer (TCCTCCGCTTATTGATATGC). The PCR thermal cycling conditions were as follows: initial denaturation at 95 °C for 3 min, followed by 35 cycles of denaturation at 95 °C for 30 s, annealing at 55 °C for 30 s, and extension at 72 °C for 45 s, with a final extension at 72 °C for 5 min. The resulting sequences were processed through denoising, error correction, and demosaicing using the UNOISE3 algorithm (with parameters set to unoise_alpha = 2 and minsize = 8, following default settings), which generated zero-radius operational taxonomic units (ZOTUs). Fungal ZOTUs were quantified and taxonomically classified by aligning with the UNITE and SILVA (v138) databases. Alpha and beta diversity metrics were calculated to assess the prokaryotic microbial communities. All samples met quality control standards, with a base recognition accuracy of 99.9%. For high-throughput sequencing analysis, a total of 16 biological samples were collected (4 from each of the three treatment groups: sedimentation, biofilm, shellfish, and macroalgae), with each group consisting of 4 biological replicates. DNA extraction and 16S rRNA/ITS sequencing were performed on each biological replicate individually. Technical replicates were not performed. Bioinformatics analysis of the sequencing data was conducted using USEARCH (version 11.0.667_I86).

### 2.4. Statistical Analysis

Diversity indices and nutrient content analyses were performed using GraphPad Prism 9. Principal coordinates analysis (PCoA) based on Bray–Curtis dissimilarity was visualized using the ‘ggpubr’ package in R (version 4.4.3). The abundance of the fungal community was illustrated with a ternary plot generated by the ‘ggtern’ package (version 3.4.2). Fungal functional groups were identified and classified using the FUNGuild database. Redundancy analysis (RDA) was conducted using the ‘rda’ function, with ordination performed via ‘cmdscale’. Forward selection of explanatory variables was achieved using the ‘ordistep’ function within the ‘vegan’ package (version 2.6-10). The final RDA visualizations were created using ‘ggplot2’ (version 3.5.0) to ensure a clear and interpretable graphical representation.

## 3. Results

### 3.1. Fungal Community Composition

**Figure 2:**
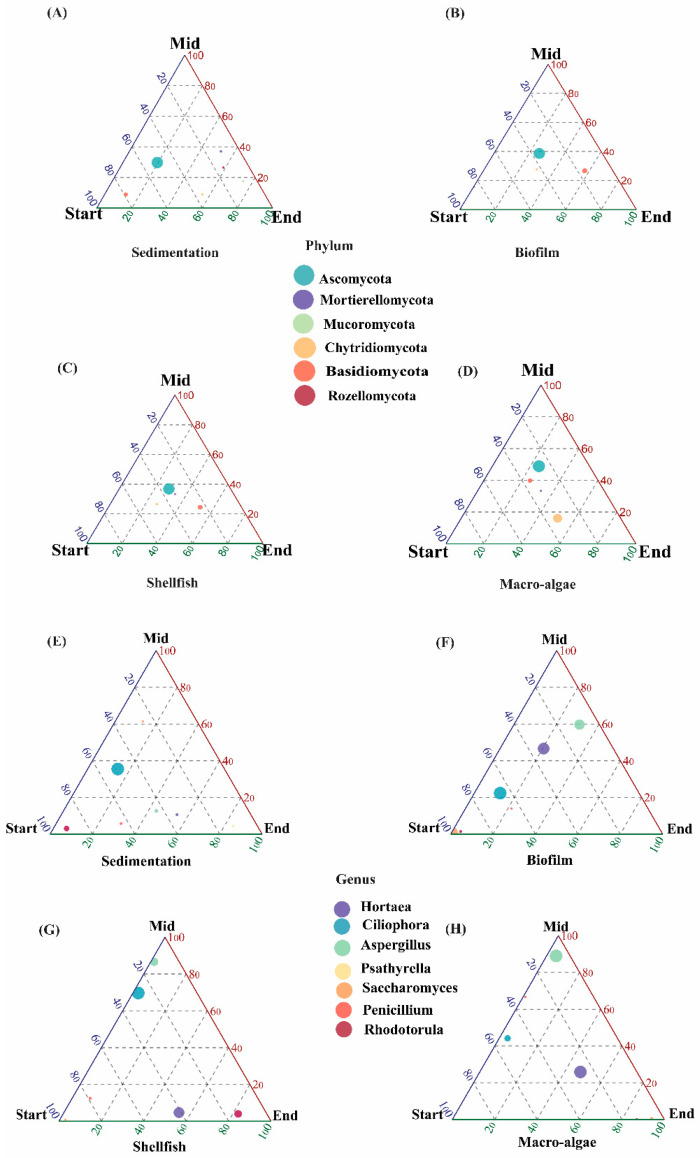
The ternary plot illustrating the relative abundance of fungal communities at the phylum (**A**–**D**) and genus levels (**E**–**H**) across different treatments at different sampling times.

The relative abundance of dominant fungi under different treatments is shown in Figure 2. At the phylum level, Ascomycota, Basidiomycota, and Chytridiomycota, along with other phyla, were detected in various areas. *Ascomycota* was the most abundant across all areas, while *Basidiomycota* and *Chytridiomycota* were present in smaller amounts. The abundance of *Ascomycota* increased progressively from the sedimentation area to the macroalgae area, with the highest abundance observed in macroalgae. The fungal composition was further analyzed, particularly at the genus level. Among the seven most abundant genera, three are the most dominant: *Hortea*, *Ciliphora,* and *Aspergillus*. *Ciliphora* is the most abundant in the sedimentation area. In the biofilm area, at the start, *Ciliphora* is the most abundant, while in the middle of the experiment, *Hortea* and *Aspergillus* are shown as the most abundant. In the shellfish area, at the start of the experiment, the abundance of Aspergillus appeared low, while *Ciliphora* was the most abundant. At the end of the experiment, *Hortea* appeared to be the most abundant. In the macroalgae unit, *Ciliphora* abundance was low compared to the shellfish area, while the abundance of the *Aspergillus* and *Hortea* was significantly increased (*p* < 0.05).

### 3.2. Dynamics of the Fungal Community

**Figure 3:**
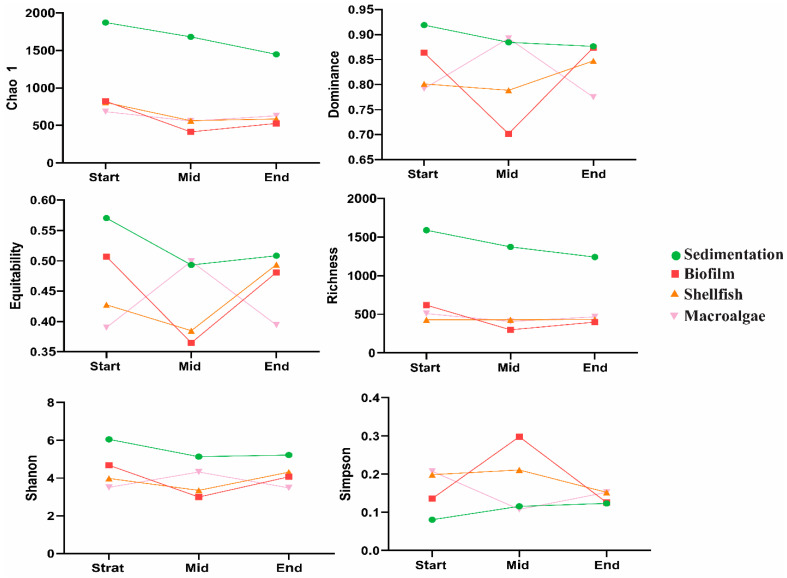
The α diversity of the fungi community (mean ± SD, *n* = 4) over treated units at different sampling times.

The alpha diversity of the water fungal community under different treatments is presented in Figure 3. Chao1 richness was highest in the sedimentation units, showing a significantly higher value compared to other areas (*p* < 0.01). Chao1 values were initially high in the sedimentation area but declined towards the end, while they remained stable in other areas. The dominance index was higher in the sedimentation area at the beginning. However, in the middle stage, dominance levels increased in both the sedimentation and macroalgae areas, becoming comparable. Conversely, the dominance concentration significantly decreased during the middle stage in other areas but later increased to levels observed in the sedimentation and macroalgae areas. In the shellfish area, the dominance concentration decreased further during the middle stage before significantly increasing at the end. Equitability was generally higher in all areas at the initial stage; then, it decreased in the middle and significantly increased at the end. However, equitability increased during the middle stage in the macroalgae area and declined significantly at the end (*p* < 0.01). Richness was significantly higher in the sedimentation area, remaining consistent across the other three areas. Shannon and Simpson diversity indices exhibited opposite trends: Shannon diversity was higher in all areas, whereas Simpson diversity was lower across all areas except the biofilm area. Shannon diversity increased significantly during the middle stage but decreased at the end (*p* < 0.01).

**Figure 4:**
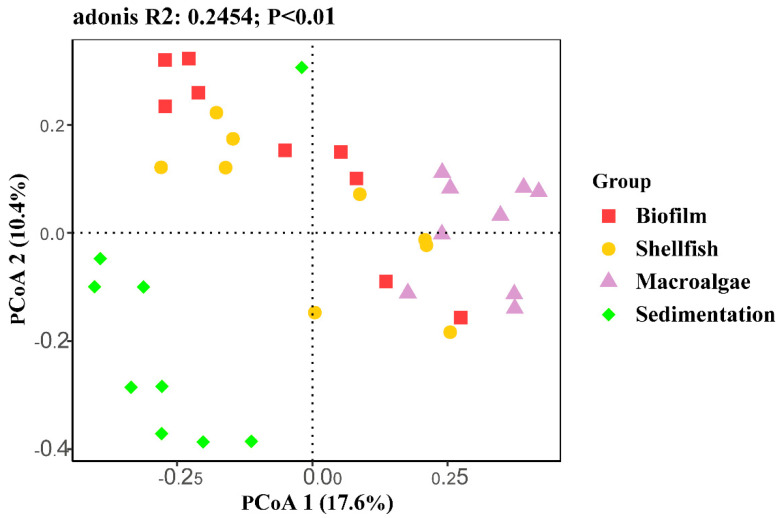
Principal coordinates analysis (PCoA) based on Bray–Curtis dissimilarity (mean ± SD, *n* = 4) of fungal community composition across different treatments.

Principal coordinate analysis (PCoA) demonstrated clustering of the 16S rRNA data (Figure 4). All downstream analyses of relative abundance were conducted using the average OTU read abundance (R^2^ = 0.2454, *p* < 0.01). PCoA revealed a distinct and recurring trajectory of the prokaryotic and eukaryotic communities, transitioning from planktonic organisms to fungal community assembly. The fungal community in the sedimentation area was notably distinct, while those in the biofilm and shellfish areas were similar. The fungal community in the macroalgae area was markedly different from those in other areas.

### 3.3. Correlation Between Nutrients and the Fungal Community

Nutrient concentrations displayed variations across different units of the system, signifying the influence of PSB and consequent shifts over time and system components (Table 1). Significant nutrient removal efficiencies were observed: COD_Mn_ stood at 71.42%, while removal rates for NO_2_^−^-N, NO_3_^−^-N, PO_4_^3−^-P, and NH_4_^+^-N were 87.20%, 88.80%, 91.72%, and 91.37%, respectively. There was a significant decrease in NO_2_^−^-N, NO_3_^−^-N, and PO_4_^3−^-P between the shellfish and macroalgae areas (*p* < 0.05). The removal of COD from the shellfish area was significant (*p* < 0.05), reaching a minimum level of 1.28 mg L^−1^.

**Figure 5:**
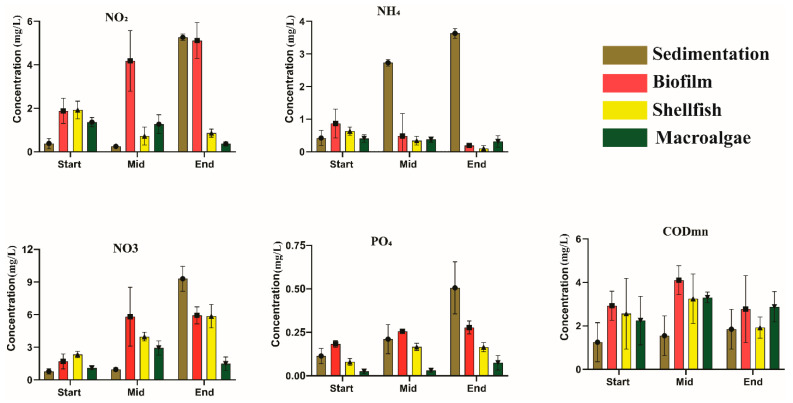
Treatment efficiencies of the nutrients (mean ± SD, *n* = 4) in the wastewater bioremediation system across different treatments.

The nutrient concentrations in different areas at various periods are shown in Figure 5. The concentrations of NH_4_^+^-N, NO_3_^−^-N, NO_2_^−^-N, and PO_4_^−3^-P and CODₘₙ differed significantly from the start to the end of the experiment (*p* < 0.05). At the start of the experiment, the concentration of NO_2_^−^-N was low in the sedimentation area but higher in the other areas. As the experiment progressed, the concentration of NO_2_^−^-N increased significantly compared to the initial values. By the end of the experiment, NO_2_^−^-N concentration was significantly higher in the sedimentation and biofilm areas (*p* < 0.05), whereas it was significantly lower in the macroalgae area (*p* < 0.05). The concentration of NH_4_^+^-N was almost uniform across all areas at the start of the experiment. However, as the experiment proceeded, NH_4_^+^-N concentrations began to decrease. By the end, they were significantly lower than at the beginning in all areas except the sedimentation area, where NH_4_^+^-N concentration was significantly higher (*p* < 0.05).

**Figure 6:**
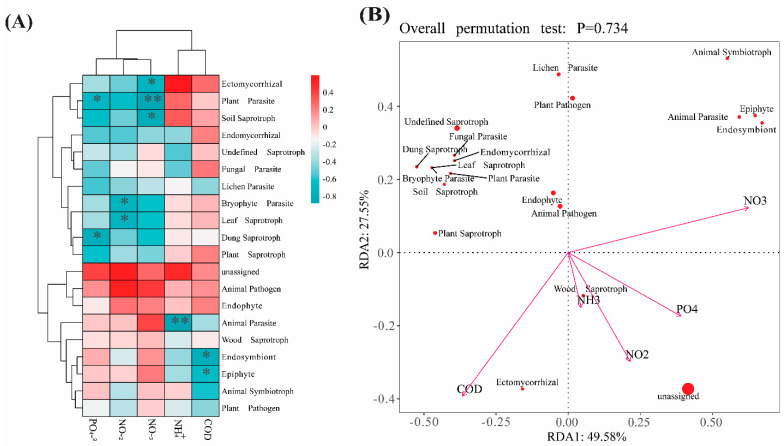
A correlation heatmap between the fungal function and nutrients (**A**); redundancy analysis of nutrients and the fungal function (**B**). (mean ± SD, *n* = 4). * significant *p* < 0.05, ** very significant *p* < 0.01.

NO_3_^−^-N concentrations were low during the initial phase of the experiment. In the middle phase, concentration increased significantly except in the sedimentation area (*p* < 0.05). By the end of the experiment, NO_3_^−^-N concentrations were significantly higher in all areas except the macroalgae area, where they were significantly lower (*p* < 0.05). At the start of the experiment, PO_4_^−3^ concentrations were below 0.25 mg/L and were relatively consistent across all areas. These levels remained stable during the middle phase of the experiment. However, by the end of the experiment, PO_4_^−3^ concentrations were significantly higher in the sedimentation and biofilm areas (*p* < 0.05). To explore the relationship between nutrients and fungal functional groups, it is evident that NO_3_^−^-N is significantly negatively correlated with soil saprotrophs, ectomycorrhizal fungi, and plant parasites. NH_4_^+^-N is significantly negatively correlated with animal parasites, and COD_Mn_ is significantly negatively correlated with endosymbionts and epiphytes. PO_4_^−3^-P is significantly negatively correlated with dung saprotrophs and plant parasites (*p* < 0.05) (Figure 6).

## 4. Discussion

### 4.1. Community Composition

The fungal community identified in the wastewater study showed significant potential for removing organic pollutants. Additionally, the findings suggest that wastewater could be a valuable source for isolating diverse fungal groups, which can be further studied and characterized for their role in degrading emerging pollutants in ecosystems. Ascomycota, the largest phylum within both aquatic and terrestrial fungal kingdoms, comprises over 65% of known fungi and plays a critical role in pollutant transformation [30] [31]. An integrative analysis of fungal communities involved in aquaculture wastewater bioremediation is essential for identifying specific fungal traits associated with enhanced decontamination efficiency. Such insights can be utilized to optimize existing bioremediation strategies and develop novel approaches [32]. Fungi are capable of utilizing a wide range of organic substrates for growth and play a significant role in elemental cycles, including the reduction of nitrogen and turnover in the carbon cycle [33]. The dynamics and diversity of fungal populations in wastewater treatment systems are critical and cannot be overlooked [34]. This study investigates the influence of photosynthetic bacteria (PSB) on nutrient removal, as well as the abundance, diversity, and composition of fungal communities in integrated aquaculture wastewater treatment systems (IAWBS). The most abundant fungal phylum identified in this study was Ascomycota, which was predominant across all sampled areas. This was followed by Mortierellomycota and Basidiomycota, as illustrated in Figure 2A–D. The composition of the fungal community aligns with observations from intertidal zones, mudflats, and sediments [35,36]. Notably, the relative abundance of Ascomycota among eukaryotes is of particular significance.

Ascomycota and Chytridiomycota are capable of secreting cellulase, enabling them to degrade cellulose and treat cellulose-containing wastewater effectively. A study on aerobic sludge revealed a high abundance of Ascomycota, which was linked to the addition of cellulose-rich substrates in membrane reactors [37]. Within Ascomycota, the class Sordariomycetes is particularly widespread, encompassing many microfungi that are ubiquitous and cosmopolitan. These fungi can exist as pathogens or endophytes in plants and animals, including arthropods and mammals [38]. In this study, the most abundant genera identified were *Hortea*, *Aspergillus*, and *Ciliphora*. The genus *Aspergillus*, a filamentous fungus, is widely utilized in bioremediation processes, particularly for treating wastewater contaminated with heavy metals, petroleum, and other pollutants. In this study, *Aspergillus* and *Hortea* were identified as the dominant genera within the fungal community in the shellfish area, with their abundance increasing following the addition of the PSB in the system (Figure 3). These findings align with those the authors of [39], who reported that during the cultivation of *Litopenaeus vannamei, Aspergillus* appeared to be the most dominant genus among the fungal communities. As saprophytic microorganisms, fungi acquire nutrients by decomposing organic matter [27]. Throughout the study, the accumulation of uneaten shrimp feed, feces, and dead microorganisms in the bio-floc led to increased organic matter concentrations, creating favorable conditions for fungal growth. *Aspergillus,* along with some other fungi, has been shown to improve the water quality in aquaculture systems [29]. Additionally, they may contribute to the bio-flocculation process, enhancing the formation of larger and more stable bio-flocs [27]. The frequent presence of *Aspergillus* species in shrimp ponds, as reported by [40], further supports these observations. This is likely due to the potential of *Aspergillus* to act as a significant producer of bio-flocculants, underscoring its ecological and functional importance in aquaculture systems. Additionally, the genus *Aspergillus* has shown potential probiotic properties in aquaculture, contributing to nutrient removal, enhanced immunity, improved growth performance, regulation of gut microbiota, and hematological parameters [41]. Thus, the addition of photosynthetic bacteria (PSB) in the shellfish area may enhance nutrient removal by stimulating the growth of fungal species.

At the genus level, *Hortaea*, a halophilic fungus [42], is commonly found in seawater and shellfish environments. In contrast, *Candida* is the most abundant genus in biofilm areas. *Candida* has the ability to form biofilms on various artificial substrates, with a preference for materials such as plastics, silicone, and metals commonly used in medical and industrial settings [43]. Water pollution is one of the most widespread and detrimental forms of environmental contamination. Microorganisms play an indispensable role in various wastewater treatment methods. The microbial treatment and purification of sewage primarily involves a material cycling process within the treatment system, driven by the synergistic interactions of microorganisms with nutrients [44].

### 4.2. Diversity Indices and Community Composition Shifts

The investigation of fungal communities in aquatic environments is gaining increasing attention due to the emergence of advanced molecular techniques, such as next-generation sequencing (NGS), which have revealed unexpected levels of abundance, ecological functions, interactions, and unresolved phylogenetic relationships [45]. A lot of studies have been reported on the bacterial communities [46]; however, research on fungal biodiversity in integrated aquaculture wastewater treatment systems (IAWBS) remains limited, with most studies focusing primarily on activated sludge.

Diversity indices were calculated to evaluate the within-sample complexity of individual fungal populations, as detailed in Figure 3. This figure illustrates the Shannon and Chao indices, which are representative measures of community diversity and richness, respectively. The Shannon index increased significantly, from 3.8 to 4.0 in macroalgae and from 3.8 to 3.9 in the shellfish area. Similarly, the Chao index rose significantly from 700 to 1000 following the addition of photosynthetic bacteria (PSB). These results suggest that PSB enhances the diversity and richness of microbial communities, consistent with previous findings [47].

The Simpson index across all samples showed minimal variation, ranging from 0.757 in the influent to 0.940 in the effluent. The highest Shannon diversity (H) was observed in the effluent (3.52), while the lowest was recorded in the influent (2.163) [20]. The Shannon and Simpson indices reported in this study were higher than those in previous research by the authors of [48] who documented Shannon index values ranging from 1.32 to 2.29 and Simpson index (1-D) values ranging from 0.54 to 0.81. Similarly, studies on fungal communities in activated sludge samples reported Shannon index values ranging from 0.1 to 2.51 and Simpson index values ranging from 0.05 to 0.95 [38,46] which are generally lower than the values obtained in this study.

### 4.3. Fungi Relationships with Nutrients

Bioremediation has increasingly been recognized as an effective and sustainable strategy for enhancing water quality in aquaculture effluents and mitigating potential environmental pollution. For the sustainability of aquaculture systems and water resources, the efficient treatment and management of nitrogenous compounds in wastewater are essential. Fungi, in particular, have demonstrated the ability to convert ammonium (NH_4_^+^-N) into nitrites (NO_2_^−^-N) and nitrates (NO_3_^−^-N) through nitrification [29]. Our redundancy analysis (RDA) and functional guild analysis (Figure 5) revealed that chemical oxygen demand (COD_Mn_), NH_4_^+^-N, NO_3_^−^-N, NO_2_^−^-N, and PO_4_^−3^-P exhibited a negative correlation with specific fungal functional groups, including ectomycorrhizal fungi, plant pathogens, dung saprotrophs, and animal pathogens. This suggests a close relationship between nutrient concentrations and fungal community composition. Environmental factors play a significant role in shaping microbial diversity patterns, as previously suggested [49]. Fungi have the ability to remove many harmful pollutants from the wastewater [50], and their denitrification capabilities can exceed those of bacteria [51]. Nutrients such as NO_2_^−^-N, NH_4_^+^-N, and NO_3_^−^-N can influence the trophic status of aquatic environments and impact the structure of eukaryotic microbial communities [52]. In our study, the concentrations of NO_2_^−^-N, NO_3_^−^-N, NH_4_^+^-N, and PO_4_^−3^-P decreased significantly in the shellfish and macroalgae areas, indicating that adding (PSB) in these areas altered the fungal community structure, enhancing nutrient removal (*p* < 0.05). The removal rates of these (NO_2_^−^-N, NO_3_^−^-N, NH_4_^+^-N, PO_4_^−3^-P, and COD_Mn_) nutrients were 87.20%, 88.20%, 91.37%, 91.72%, and 71.42%, respectively (Figure 4). Nitrogen reduction systems, which are based on fungi, have the potential to replace the conventional biological treatment method for nutrient removal. Fungi regulate nutrient concentrations and facilitate the removal of nitrogenous compounds [53,54]. It was found that plant saprotrophs, ectomycorrhizal and bryophyte parasites, and leaf saprotrophs were significantly negatively correlated with NO_3_^−^-N and NO_2_^−^-N by exploring the relationship between fungal functional groups and nutrients. (Figure 6). According to the analysis of fungi and microorganisms, *Candida* was significantly positively correlated with nitrate respiration and nitrogen respiration. *Candida* was significantly positively correlated with nitrite respiration, denitrification, nitrous oxide denitrification, nitrate denitrification, and nitrite denitrification. *Nemania* was significantly positively correlated with nitrification (Figure 6B).

**Figure 7:**
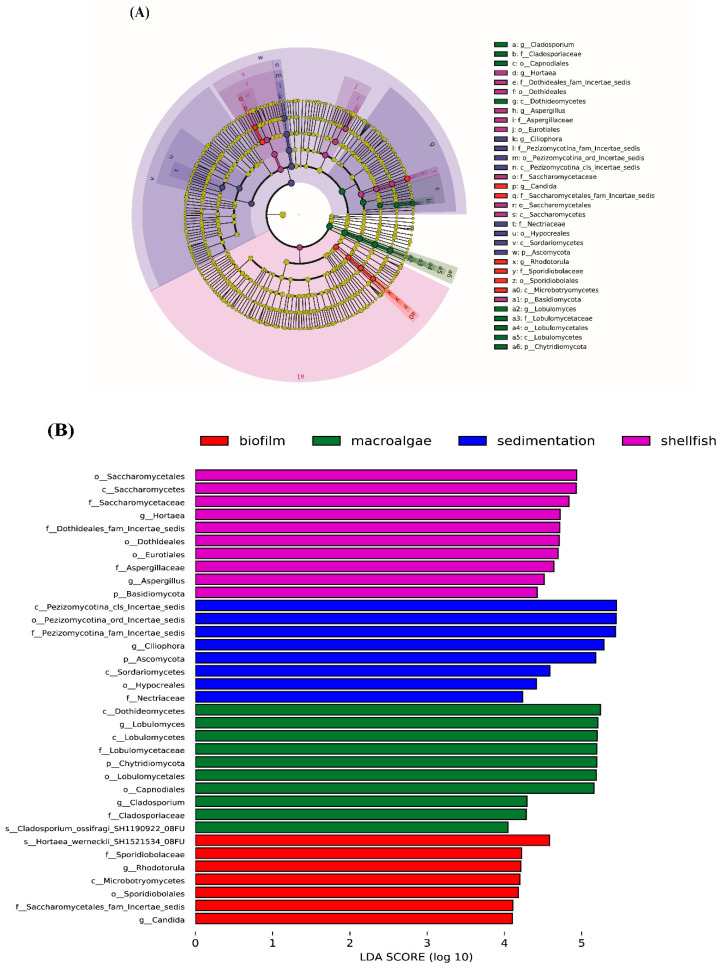
(**A**) The LEfSe cladogram representing the distribution of statistically significant taxa (biomarkers) from phylum to genus level under four treatment areas. (**B**) LDA scores histogram identifies clades among the detected taxa with a statistical and biological difference between the communities at a threshold value of 4 (mean ± SD, *n* = 4).

Our LEfSe and LDA analyses identified potential fungal biomarkers at the genus level. In sediment samples, *Ciliophora* dominated, while *Rhodotorula* and *Candida* were prevalent in biofilm units. In the shellfish area, *Hortaea* and *Aspergillus* were dominant, whereas *Lobulomyces* and *Cladosporium* were predominant in macroalgae areas (Figure 7). These findings align with previous studies that identified various fungi involved in organic matter conversion in wastewater, including *Ciliophora* and *Penicillium* [55]. *Rhodotorula* is involved in COD_Mn_ removal and the metabolism of polycyclic aromatic hydrocarbons (PAHs) and nitrogenous compounds [56]. Members of the class Saccharomycetes, such as *Candida* and *Saccharomyces*, contribute to environmental nitrogen removal through denitrification, degradation of phenols and hydrocarbons, and the removal of heavy metals and other pollutants like chromium [56,57,58]. *Cladosporium* exhibits diverse roles in plant growth promotion, biocontrol, and disease-causing activities [59]. Dominant and rare fungal genera identified, including members of the classes Sordariomycetes (*Fusarium*) and Eurotiomycetes (*Aspergillus*, *Penicillium*), can transform organic contaminants like nitrogenous and phosphorus compounds present in wastewater [34,60,61]. Enhanced nitrogenous compound removal by *Penicillium* in activated sludge systems has also been reported [62]. Genera such as *Fusarium* and *Trichoderma* have demonstrated denitrification abilities [63], while other fungi within this division can transform xenobiotics [34,64]. The fungal community structure observed in this study suggests that microbial communities contribute significantly to the acclimation and adaptation capacity of IAWBS to environmental changes. The absence of PSB resulted in reduced fungal community diversity, whereas its presence in combination with shellfish and macroalgae not only altered the fungal community composition but also enhanced fungal diversity. Ultimately, the IAWBS improved water quality by effectively reducing nutrient concentrations. From an integrated perspective, the comparative analysis of the treatments reveals significant differences in feasibility, economic viability, productivity, and real-world applicability. The treatment combining photosynthetic bacteria (PSB) and native fungal communities demonstrated superior nutrient removal efficiency, suggesting a more sustainable and ecologically balanced approach to wastewater remediation. Unlike chemical treatments or mechanical filtration systems, the use of microbial consortia is cost-effective, requiring minimal energy input and infrastructure investment. The enhanced pollutant removal observed in this treatment implies that PSB and fungi not only contribute individually but also act synergistically to improve system performance [26]. Economically, the production and application of PSB—particularly strains like *Rhodopseudomonas palustris*—are relatively inexpensive and can be scaled using low-cost fermentation techniques. Fungi, often naturally present or introduced through organic substrates, further reduce operational costs. When compared to treatments lacking microbial additions or relying solely on one microbial group, the integrated approach proved more efficient without significantly increasing maintenance requirements or resource consumption [65]. In terms of productivity, the improvement in water quality directly supports better aquaculture output by reducing the stress and disease burden on cultured species. This increases survival rates and growth performance, adding downstream economic benefits for farmers. Furthermore, the practical application of such systems is highly feasible for small- to medium-scale aquaculture operations, particularly in regions where regulatory pressure on effluent discharge is increasing [66]. Thus, integrating PSB and fungal communities offers a promising, low-cost, and scalable solution for aquaculture wastewater management, supporting both environmental sustainability and economic resilience. Future studies should focus on system optimization, such as inoculation timing and dosage, and assess long-term field applicability across different aquaculture species and environmental conditions.

## 5. Conclusions

Our study demonstrates that photosynthetic bacteria (PSB) exert a significant impact on the composition of fungal community structure within aquaculture wastewater. The introduction of PSB not only altered the overall fungal community composition but also induced notable shifts in the dominant fungal taxa. The fungal community was primarily composed of the phyla *Ascomycota* and *Chytridiomycota*, with *Aspergillus* and *Ciliphora* identified as the dominant genera in the shellfish and macroalgae areas. Furthermore, our analysis revealed significant negative correlations between nutrient concentrations (COD_Mn_, NH_4_^+^-N, NO_3_^−^-N, NO_2_^−^-N, and PO_4_^−3^-P) and specific fungal functional groups, including epiphytes, animal pathogens, dung saprotrophs, plant pathogens, and ectomycorrhizal fungi. These findings highlight the pivotal role of PSB in modulating fungal communities and suggest potential pathways through which nutrient dynamics influence fungal functional groups in integrated aquaculture wastewater bioremediation. The broader implication of this work lies in its potential to inform the development of sustainable and low-cost bioremediation technologies for aquaculture systems. Integrating microbial consortia like PSB and fungi offers an environmentally friendly alternative to chemical treatments, contributing to more circular and regenerative water use practices in coastal and inland aquaculture.

However, several scientific gaps remain. The specific mechanisms underlying the synergistic interactions between PSB and fungi at the molecular and metabolic levels are still unclear. In particular, the role of interspecies signaling, substrate competition, and niche differentiation warrants deeper investigation. Future research should focus on metatranscriptomic and metabolomic analyses to elucidate functional interactions between bacteria and fungi. Long-term field-scale evaluations are needed to assess the stability and scalability of such integrated systems under varying environmental conditions.

## Figures and Tables

**Table 1:** Nutrient removal efficiency rate in IAWBS.

Parameter (mg L^−1^)	Sedimentation	Biofilm	Shellfish	Macroalgae	Removal Efficiency (%)
NO_2_^−^-N	0.422 ± 0.23 ^b^	0.076 ± 0.0534 ^a^	0.464 ± 0.0566 ^b^	0.054 ± 0.0534 ^a^	87.20
NO_3_^−^-N	13.302 ± 1.15 ^c^	5.935 ± 0.778 ^b^	5.865 ± 1.077 ^b^	1.489 ± 0.6111 ^a^	88.80
PO_4_^3−^-P	0.906 ± 0.15 ^c^	0.278 ± 0.0378 ^b^	0.165 ± 0.0260 ^b^	0.075 ± 0.0141 ^a^	91.72
NH_4_^+^-N	3.630 ± 0.15 ^b^	0.191 ± 0.0567 ^a^	0.106 ± 0.0898 ^a^	0.313 ± 0.1759 ^a^	91.37
COD_Mn_	4.48 ± 0.277 ^b^	3.36 ± 0.666133 ^b^	1.28 ± 0.48 ^a^	2.56 ± 0.2444 ^ab^	71.42

Notes: Different superscript letters in the same lines symbolize significant (*p* < 0.05, one-way ANOVA) differences among the different sampling units.

## Data Availability

The raw data supporting the conclusions of this article will be made available by the authors on request.
